# Estimating the Prevalence of Malignant Hyperthermia Susceptibility Based on Causative *RYR1* and *CACNA1S* Variants

**DOI:** 10.3390/ijms27188327

**Published:** 2026-09-19

**Authors:** Ekaterina Efremova, Elena Baranova, Anton Esibov, Anastasiia Rozhkova, Ekaterina Rutkovskaya, Julia Krupinova, Olga Mityaeva, Mary Woroncow, Sherzod Abdullaev, Karin Mirzaev, Viktor Bogdanov, Pavel Volchkov, Dmitry Sychev

**Affiliations:** 1 Petrovsky National Research Center of Surgery, Moscow 119435, Russia; baranova.gen@gmail.com (E.B.); abdullaevsp@gmail.com (S.A.); karin05doc@yandex.ru (K.M.); dimasychev@mail.ru (D.S.); 2 Russian Medical Academy of Continuous Professional Education, Moscow 125993, Russia; 3 Federal Research Center for Innovator and Emerging Biomedical and Pharmaceutical Technologies, Moscow 125315, Russia; esibov_aa@academpharm.ru (A.E.); rozhkova_av@academpharm.ru (A.R.); rutkovskaya_ea@academpharm.ru (E.R.); j.krupinova@gmail.com (J.K.); mityaeva_on@academpharm.ru (O.M.); bogdanov_vp@academpharm.ru (V.B.); volchkov_py@academpharm.ru (P.V.); 4 Moscow Center for Advanced Studies, Moscow 123592, Russia; 5 Loginov Moscow Clinical Scientific Center, the Moscow Healthcare Department, Moscow 111123, Russia; 6 Faculty of Fundamental Medicine, Lomonosov Moscow State University, Moscow 119991, Russia; innovations.endo@yandex.ru

**Keywords:** malignant hyperthermia, susceptibility, *RYR1*, *CACNA1S*, genetic variants, variant frequencies

## Abstract

Malignant hyperthermia susceptibility (MHS; OMIM 145600, 601887) is a pharmacogenetic condition in which exposure to volatile anesthetics and/or succinylcholine can trigger a potentially life-threatening hypermetabolic crisis of skeletal muscle. MHS is predominantly associated with pathogenic variants in *RYR1*, whereas *CACNA1S* contributes a substantially smaller proportion of genetically confirmed cases. Estimating the population prevalence of MHS is challenging because manifestation requires exposure to a triggering agent, penetrance is incomplete, and the evidentiary strength supporting individual variants varies considerably. In this study, MHS-associated *RYR1* and *CACNA1S* variants were compiled from four complementary resources—EMHG, ClinGen MHS VCEP, HGMD, and ClinVar—and subjected to source-specific pathogenicity and phenotype filtering. A total of 13,918 unique reported variants were initially identified. Progressive evidence-based curation generated three nested variant tiers: a high-confidence Expert tier comprising 102 variants; an expanded HGMD-inclusive tier comprising 446 variants; and a full expanded ClinVar-inclusive tier comprising 593 variants. Population allele frequencies were evaluated using gnomAD v4.1.0 and the Russian GDB v1.3.4 database. The high-confidence Expert tier yielded genetically inferred MHS frequencies of approximately 1 in 807 in gnomAD and 1 in 1078 in GDB. Inclusion of HGMD-derived variants increased the corresponding estimates to approximately 1 in 50 and 1 in 104, respectively.

## 1. Introduction

Malignant hyperthermia (MH) is a rare but potentially fatal complication of general anesthesia, caused by uncontrolled calcium release from the sarcoplasmic reticulum of skeletal muscle. Clinical manifestations of MH include acidosis, hypercapnia, tachycardia, hyperthermia, muscle rigidity, compartment syndrome, rhabdomyolysis with subsequent elevation of serum creatine kinase (CK), hyperkalemia with the risk of cardiac arrhythmia or even cardiac arrest, and myoglobinuria with the risk of renal failure. In almost all patients, the first signs of MH (hypercapnia, tachycardia, and tachypnea) appear in the operating room; however, symptoms may also emerge during the early postoperative period [1]. For anesthesiologists and intensivists, MH is of particular practical importance, since timely recognition of the risk allows triggering agents to be avoided and safe anesthetic protocols to be used.

Estimating the prevalence of MHS is more difficult than estimating the frequency of MH crises themselves. A clinical crisis occurs only when genetic predisposition, exposure to a triggering agent, and sufficient individual susceptibility coincide. As a result, the reported frequency of MH episodes is inevitably lower than the genetic frequency of MHS. Additional reasons for this discrepancy include incomplete penetrance, variable expressivity, the absence of prior exposure to volatile anesthetics in some carriers, difficulties in diagnosing mild or atypical episodes, and the limited availability of contracture testing and functional variant validation.

The exact incidence of MH is unknown because of incomplete ascertainment of abortive forms of the syndrome. According to various sources, the incidence in children is approximately 1 in 3000–15,000 general anesthetics, and in adults approximately 1 in 50,000–100,000 general anesthetics, whereas the estimated genetic prevalence may be as high as 1 in 2000–3000 in the general population [2,3,4].

The central hypothesis of this article is that the carrier frequency of pathogenic and likely pathogenic *RYR1* variants associated with MHS exceeds the clinical frequency of reported MH crises [5]. This discrepancy should be regarded not as a contradiction but as an expected consequence of the pharmacogenetic nature of MHS: the genetic risk is present prior to exposure, whereas the clinical phenotype manifests only under specific external conditions.

### 1.1. Genetic Architecture of MHS

Variants in *RYR1* account for approximately 76% of MHS cases, whereas *CACNA1S* accounts for approximately 1% [6].

*RYR1* encodes the ryanodine receptor and represents a large calcium-release channel of the sarcoplasmic reticulum that is central to excitation–contraction coupling in skeletal muscle. In MHS, most well-established causative *RYR1* variants are missense substitutions that alter channel regulation and increase the probability of pathological calcium release in response to triggering anesthetics. This mechanism is fundamentally different from simple loss of function: for MHS, a change in the sensitivity and activation threshold of the channel is more relevant than a reduction in protein abundance [7].

Classification of *RYR1* variants is complicated by variable expressivity, reduced penetrance, and high allelic heterogeneity. The European Malignant Hyperthermia Group (EMHG) has recognized 48 *RYR1* variants as diagnostically relevant for MH [8].

*CACNA1S* encodes the alpha-1S subunit of the dihydropyridine receptor (DHPR), which is mechanically and functionally coupled to the ryanodine receptor in the skeletal muscle triad. *CACNA1S* variants may impair signal transduction from sarcolemmal depolarization to RyR1 channel opening, but their contribution to MHS is substantially smaller than that of *RYR1*. *CACNA1S* is therefore best regarded as a confirmed but minor component of the genetic architecture of MHS.

The literature also discusses other genes related to calcium homeostasis, myopathies, or rhabdomyolysis. However, a single clinical observation or biological plausibility is not sufficient to include a gene in the list of those associated with MHS. Reproducible family data, functional assays, a documented association with a positive contracture test, or a compelling statistical association are required. Otherwise, expanding the gene panel increases the proportion of variants of uncertain significance (VUS) and may lead to an overestimation of prevalence [9].

### 1.2. Criteria for Variant Causality

Current interpretation of variants associated with MHS should be based on the ACMG/AMP criteria. For *RYR1*, the work of the ClinGen Malignant Hyperthermia Susceptibility Variant Curation Expert Panel (VCEP) is particularly important, as it refines the applicability of population allele frequencies, functional data, segregation evidence, contracture-test results, and previously published lists of significant variants.

Published assessments by the ClinGen Expert Panel show that the number of *RYR1* variants that can be confidently considered causative for MHS is substantially smaller than the total number of rare variants found in population databases. In the expanded classification of 251 *RYR1* variants, only 42 were classified as pathogenic/likely pathogenic, while the majority remained in the VUS category. Based on the frequencies of these P/LP variants, a carrier frequency of approximately 1 in 300 to 1 in 1075 has been proposed, which exceeds the frequency of clinically reported MH crises [10].

In practice, variants should be divided into at least four categories: pathogenic (P), likely pathogenic (LP), variants of uncertain significance (VUS), and benign/likely benign (B/LB). For genetic-based estimates of MHS prevalence, only P/LP variants should constitute the primary analytic layer. VUS can be analyzed separately for research purposes, for example, for domain-based mapping and selection of candidates for functional testing, but they should not be included as causative variants in the primary carrier-frequency estimate [6].

This distinction is particularly important for *RYR1*, because the gene is large, harbors numerous rare missense variants, and rarity alone does not establish pathogenicity. Combining P/LP and VUS variants would artificially inflate the estimated prevalence of MHS, whereas restricting the analysis to variants with very strong functional and clinical evidence could lead to underestimation. The optimal strategy is therefore to present several tiers of evidence.

Variants in *CACNA1S* are best regarded not as alternatives to *RYR1* but as an additional layer within the same excitation–contraction coupling system. *CACNA1S* acts upstream of the ryanodine receptor in the depolarization signal-transduction pathway. Variants in this gene may therefore be biologically relevant, but their epidemiological contribution is substantially smaller.

For the final prevalence estimate, it is advisable to report the contributions of *RYR1* and *CACNA1S* separately. A combined estimate is acceptable only after filtering by identical evidence criteria; otherwise, rare VUS in the minor gene could disproportionately inflate the estimated frequency of MHS.

Missense variants are expected to predominate among MHS-causing variants. This is consistent with the pathophysiology of the disease: most causative substitutions alter the activation sensitivity of the ryanodine receptor channel, the stabilization of its closed state, or the interaction between regulatory domains. Variants leading to protein truncation (nonsense mutations), frameshift deletions/duplications, or splicing defects often result in reduced protein expression or complete loss of function (LoF) of the ryanodine receptor; in such cases MHS may occur but is not the sole manifestation. Such variants require cautious interpretation, since loss of *RYR1* function is more often associated with congenital myopathies (e.g., central core disease) than with isolated pharmacogenetic susceptibility [11,12,13].

Interpretation of these calculations should emphasize the distinction between the genetic prevalence of MHS and the clinical frequency of MH crises. A carrier of a pathogenic/likely pathogenic variant may never receive a triggering anesthetic or may not develop a recognized crisis because of incomplete penetrance. Consequently, a high genetic frequency does not imply a proportionally high frequency of observed anesthetic complications.

Mapping of pathogenic/likely pathogenic variants onto the ryanodine receptor protein should cover the following regions:

- the N-terminal regulatory region, where variants may disrupt interdomain interactions and destabilize the closed state of the channel;
- the central domain and the classical hotspot regions, historically enriched for diagnostic variants;
- the SPRY domains and solenoid regions, involved in protein–protein interactions and allosteric signal transduction;
- the C-terminal transmembrane/pore-forming region, directly linked to the channel pore and calcium release [14,15].

Causative MHS-associated variants increase the propensity of the ryanodine receptor toward pathological calcium release. Following exposure to volatile anesthetics or succinylcholine, this can lead to a sustained rise in cytosolic calcium, increased muscle contraction, and increased oxygen consumption, CO_2_ production, heat generation, and lactate production. The clinical crisis represents a late, systemic manifestation of this underlying cellular disturbance of calcium homeostasis.

In international practice, contracture tests—the in vitro contracture test (IVCT) and the caffeine–halothane contracture test (CHCT)—remain an important phenotypic standard for diagnosing MHS, particularly when the genetic result is negative or a VUS is identified. Cellular functional assays, including analysis of the calcium response to caffeine, halothane, or other stimuli, allow the effect of a specific variant to be assessed. Functional validation is especially important for novel variants or those located outside known hotspot regions, since protein position and population rarity alone do not establish causality [16,17].

## 2. Results

### 2.1. Curated MHS Variant Spectrum for Prevalence Estimation

**Figure 1:**
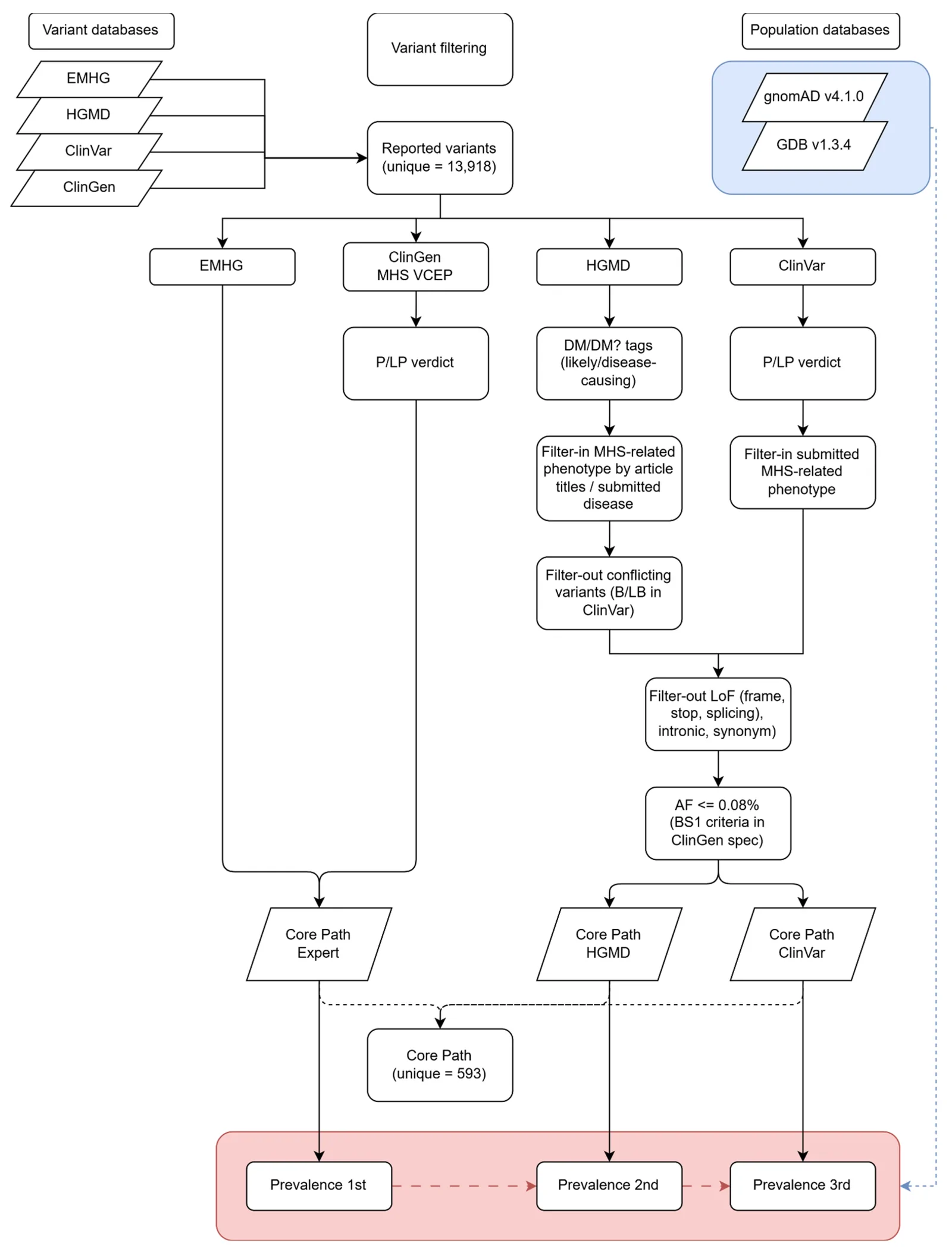
Overview of the tier-based variant framework used for prevalence estimation.

The variant curation and prevalence-estimation framework is summarized in Figure 1. Variant records were first pooled from four causative-variant databases (EMHG, HGMD, ClinVar, and ClinGen) and deduplicated to a combined set of 13,918 unique reported variants (10,584 for *RYR1* and 3334 for *CACNA1S*). This pool was then filtered along four source-specific paths, each feeding one of the three tiered variant panels used for prevalence estimation. Variants from EMHG were retained as reported, without further filtering. Variants from ClinGen were retained if they carried a pathogenic/likely pathogenic (P/LP) verdict from the ClinGen MHS Variant Curation Expert Panel (MHS VCEP); together, the EMHG and ClinGen subsets formed the Core Path Expert panel. Variants from HGMD were retained if tagged DM or DM? (disease-causing or likely disease-causing), filtered in for an MHS-related phenotype based on citing-publication titles or the submitted disease term, and filtered out if they carried a conflicting benign/likely benign (B/LB) classification in ClinVar. Variants from ClinVar were retained if they carried a P/LP verdict and filtered out if their only associated phenotype was an autosomal recessive (AR) myopathy. The HGMD- and ClinVar-derived candidates were then pooled and passed through a shared final filter, excluding loss-of-function (frameshift, stop-gain, splice-site), intronic, and synonymous variants, and retaining only variants with a population allele frequency ≤ 0.08% (the BS1 threshold specified in the ClinGen MHS VCEP criteria). Variants surviving this shared filter were attributed back to their source of origin, forming the Core Path HGMD and Core Path ClinVar panels. The three source-specific panels were subsequently merged by deduplicated union into the full Core Path reference set, and each panel independently defined one of the three nested prevalence tiers (Prevalence 1st, 2nd, and 3rd) evaluated against both population databases (gnomAD v4.1.0 and GDB v1.3.4).

**Figure 2:**
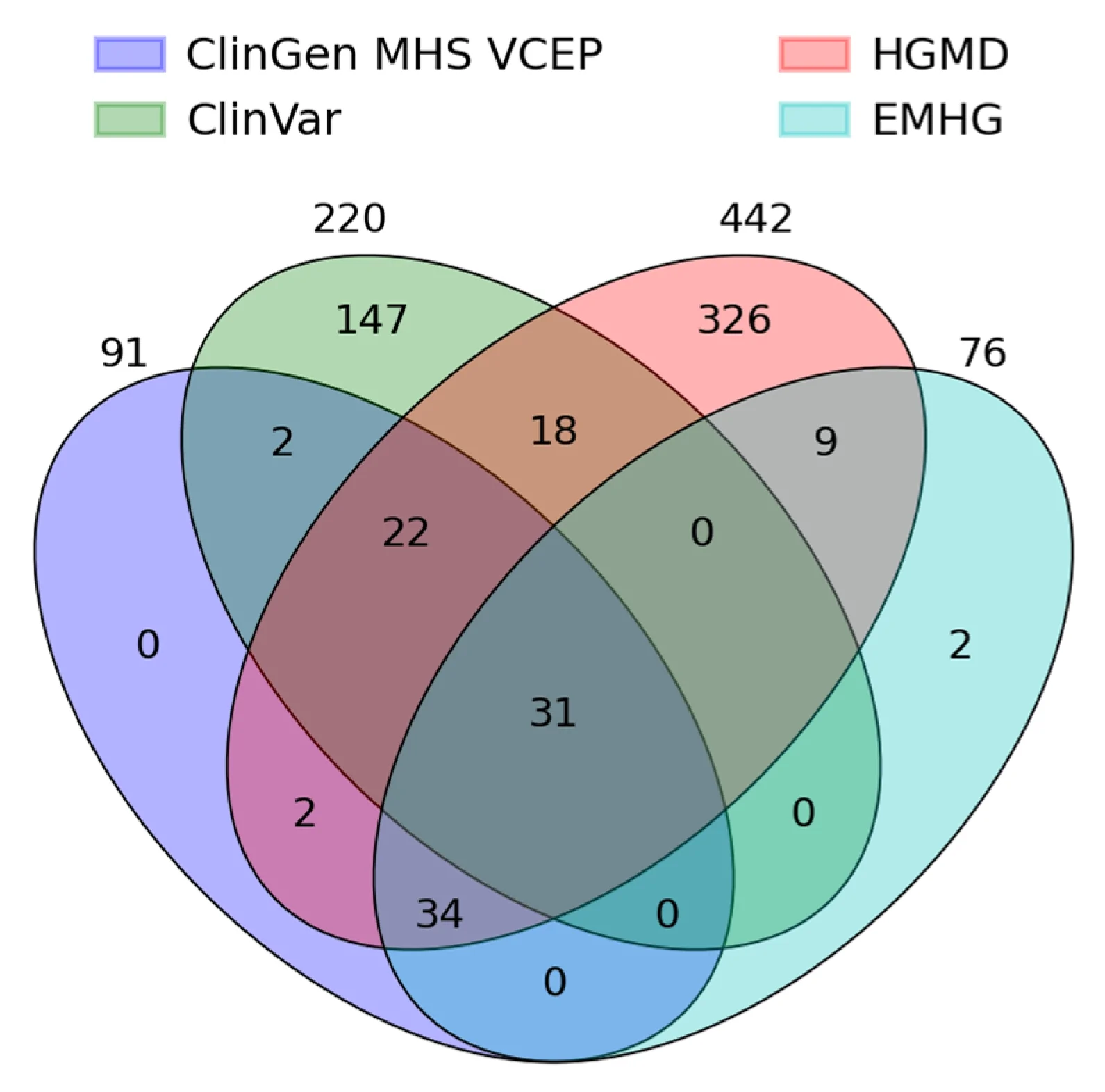
Venn diagram of the causal variant databases used in the study.

Data extraction and merging across the four source databases yielded 13,918 unique reported variants. Compilation of candidate pathogenic variants meeting each source’s own evidence criteria showed incomplete overlap between sources (Figure 2): ClinGen MHS VCEP contributed 91 variants, ClinVar 220, HGMD 442, and EMHG 76. Only 31 variants were reported by all four sources; these are listed in Supplementary Table S1. This indicates that no single database captures the full candidate pathogenic spectrum for MHS and supports the use of multiple complementary sources for variant curation.

**Figure 3:**
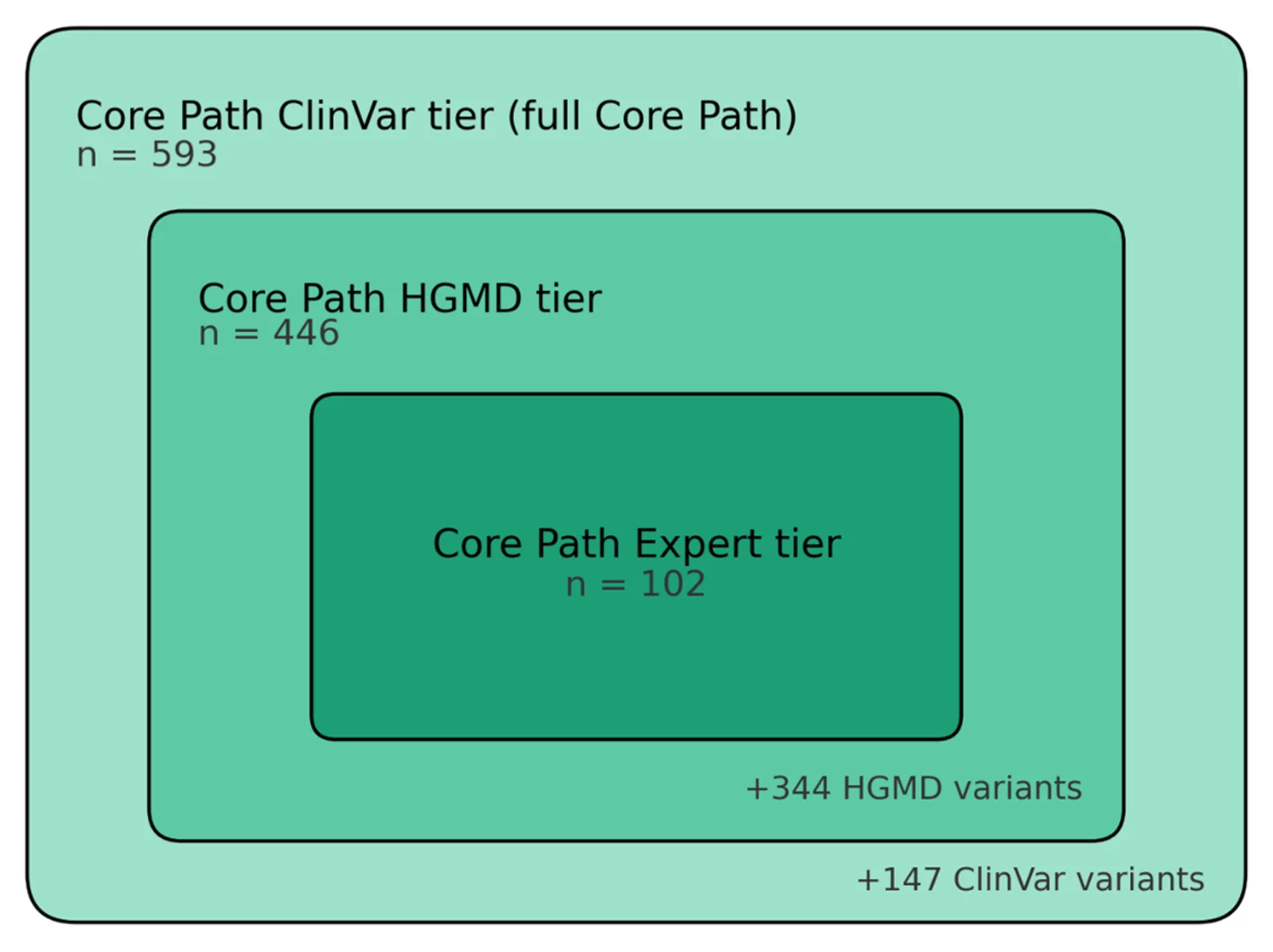
Nested composition of the Core Path variant panels used for tiered prevalence estimation.

The EMHG and ClinGen MHS VCEP subsets were combined into the Core Path Expert set, comprising 102 unique variants. Progressively taking the union of this set with the fully filtered HGMD-derived pool (442 variants meeting all HGMD-specific evidence, phenotype, and allele-frequency criteria) yielded the Core Path HGMD tier, comprising 446 unique variants. Taking the further union with the fully filtered ClinVar-derived pool (220 variants meeting all ClinVar-specific criteria) yielded the Core Path ClinVar tier, comprising 593 unique variants—the complete Core Path reference set (Figure 3).

**Figure 4:**
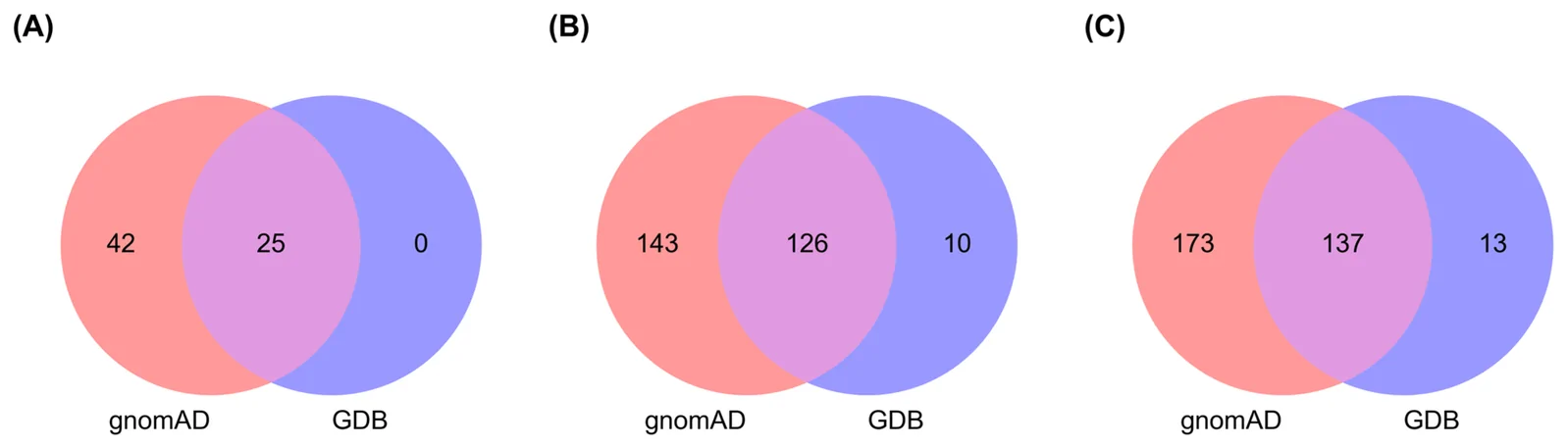
The figure shows the matching and unique variants for samples obtained from population databases (gnomAD, GDB): (**A**) Core Path Expert; (**B**) Core Path HGMD; (**C**) Core Path ClinVar.

Of the 593 curated variants, 310 (52.3%) were represented in at least one of the two population reference databases used for allele-frequency-based prevalence estimation; the allele frequencies of these 310 variants formed the basis of the prevalence calculations (Figure 4; Supplementary Table S2).

The proportion of core pathogenic MHS variants represented in each population database varied by tier and by resource, with gnomAD consistently capturing a larger share than GDB. These dataset-specific differences underscore the importance of population-matched allele-frequency data for accurate prevalence estimation.

### 2.2. Prevalence Estimates by Population and Variant Tiers

Observed allele frequencies for Core Path Expert variants were available for 25 variants in GDB and for 67 variants in gnomAD v4.1.0. The remaining Core Path Expert variants were not observed in these population datasets and therefore did not contribute to the observed allele counts used for prevalence estimation. Using the Bayesian model described in Section 4.3, conservative prevalence estimates based on the Core Path Expert set were 1 in 807 globally and 1 in 1078 in the Russian population (Table 1). The corresponding expected numbers of affected individuals were then calculated for the Russian population (146 million) and the worldwide population (8 billion), yielding approximately 135,375 affected individuals in Russia and 9,918,654 worldwide.

To evaluate the effect of variant-classification uncertainty on prevalence estimation, we next estimated prevalence across the predefined nested variant tiers (Table 2). Inclusion of HGMD-derived variants (Core Path HGMD tier) increased the estimated prevalence compared with the conservative Core Path Expert set, to 1 in 104 in Russia and 1 in 65 globally. Further expansion of the variant set to include ClinVar-derived variants (Core Path ClinVar tier, the full Core Path reference set) generated the broadest estimates, reaching 1 in 102 in Russia and 1 in 63 globally. Notably, the point estimate for the global (gnomAD-based) prevalence was numerically identical between the Core Path HGMD and Core Path ClinVar tiers, despite the ClinVar tier containing 147 additional curated variants. This is consistent with these variants being absent from gnomAD or observed at a frequency too low to shift the summed allele count at the reported level of precision. Thus, the tier-based analysis produced a prevalence range from conservative estimates based on the most stringently curated pathogenic variants to broader estimates incorporating variants of lower curation certainty. This pattern shows that prevalence estimates for MHS are highly sensitive to the variant set used for calculation.

Prevalence estimates rose sharply from the Core Path Expert tier to the Core Path HGMD and Core Path ClinVar tiers in both populations. This pattern shows that, as with other tier-based genetic prevalence frameworks, MHS prevalence estimates are highly sensitive to the variant set used for calculation.

**Figure 5:**
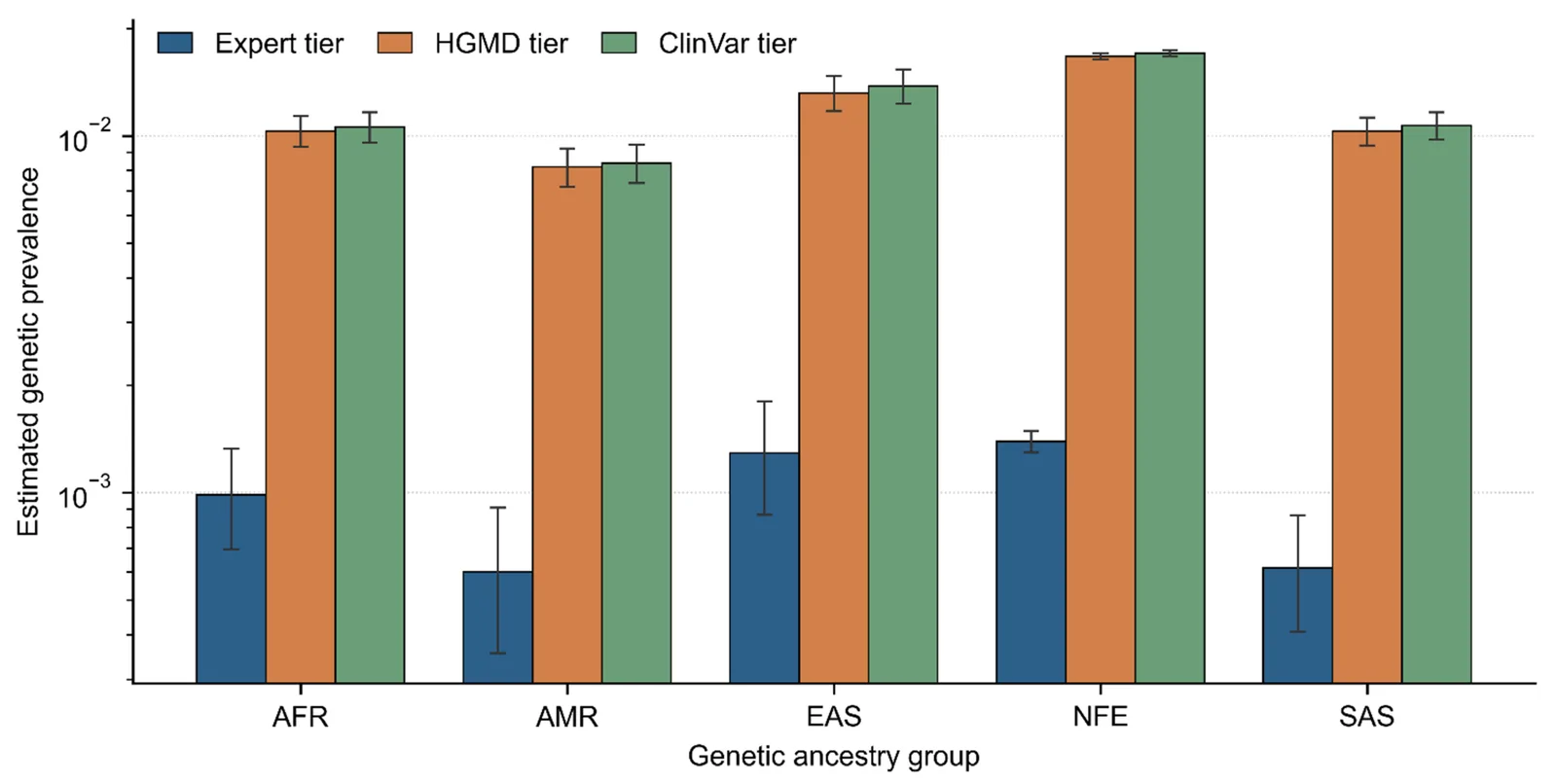
Estimated genetic prevalence of *RYR1*/*CACNA1S* malignant hyperthermia susceptibility across gnomAD v4.1 genetic ancestry groups for three variant classification tiers. Bars show point estimates; error bars denote 95% credible intervals. AFR, African/African American; AMR, Admixed American; EAS, East Asian; NFE, non-Finnish European; SAS, South Asian.

Of the 102 variants in the expert-curated tier (Core Path Expert), 67 were present in gnomAD v4.1 joint. The cumulative pathogenic allele frequency varied roughly twofold across genetic ancestry groups, being highest in non-Finnish Europeans (q = 6.96 × 10^−4^) and East Asians (6.46 × 10^−4^), and lowest in Admixed Americans (3.00 × 10^−4^) and South Asians (3.07 × 10^−4^) (Figure 5). The estimated genetic prevalence was 1 in 719 for NFE, 1 in 774 for EAS, 1 in 1015 for AFR, 1 in 1627 for SAS, and 1 in 1668 for AMR. Precision differed markedly with sample size across groups. In NFE, which contributed 1,180,054 alleles, the 95% credible interval spanned approximately ±7% of the point estimate (1 in 719, CI 672–771), whereas in EAS (AN = 44,904) and AMR (AN = 60,030) the intervals covered more than a twofold range (1 in 774, CI 555–1156 and 1 in 1668, CI 1103–2814, respectively). Consequently, the apparent between-group differences are well resolved only for NFE; for the remaining groups the intervals overlap substantially, and the rank order of estimates should not be over-interpreted. Per-ancestry estimates for the AFR, AMR, EAS, NFE and SAS groups are provided in Supplementary Table S3.

### 2.3. Population-Specific Distribution of MHS Variant Frequencies

**Figure 6:**
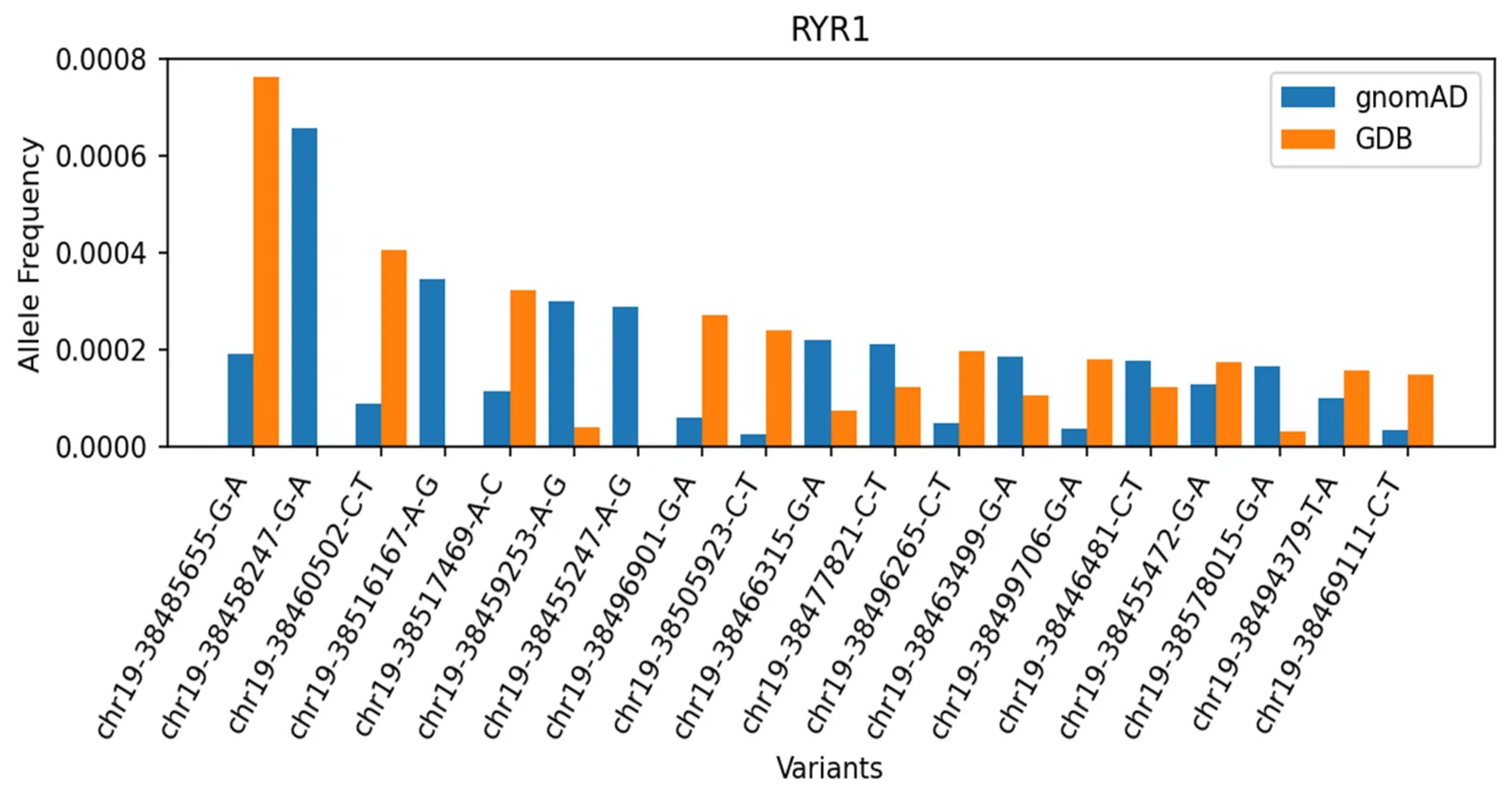
Allelic frequency for the Top 10 variants in *RYR1* in population databases (gnomAD, GDB).

**Figure 7:**
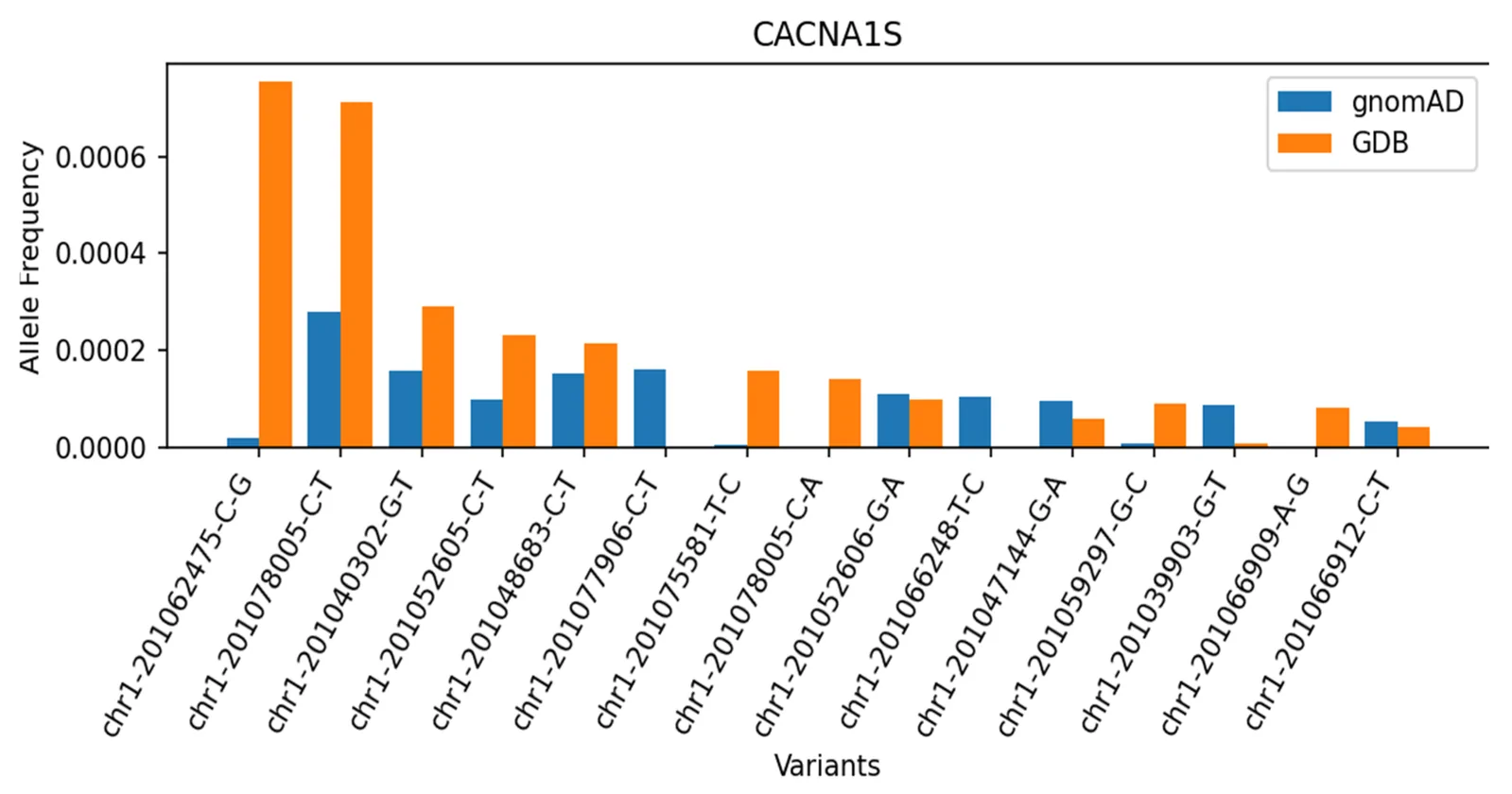
Allelic frequency for the Top 10 variants in *CACNA1S* in population databases (gnomAD, GDB).

Figure 6 and Figure 7 compare the ten most common variants in the gnomAD and Russian GDB databases for *RYR1* and *CACNA1S* genes. Notably, four of these ten variants are represented exclusively in gnomAD and are absent from GDB, which may indicate incomplete representation of causative variants within GDB.

### 2.4. Diagnostic Yield of a Prioritized Variant Panel

**Figure 8:**
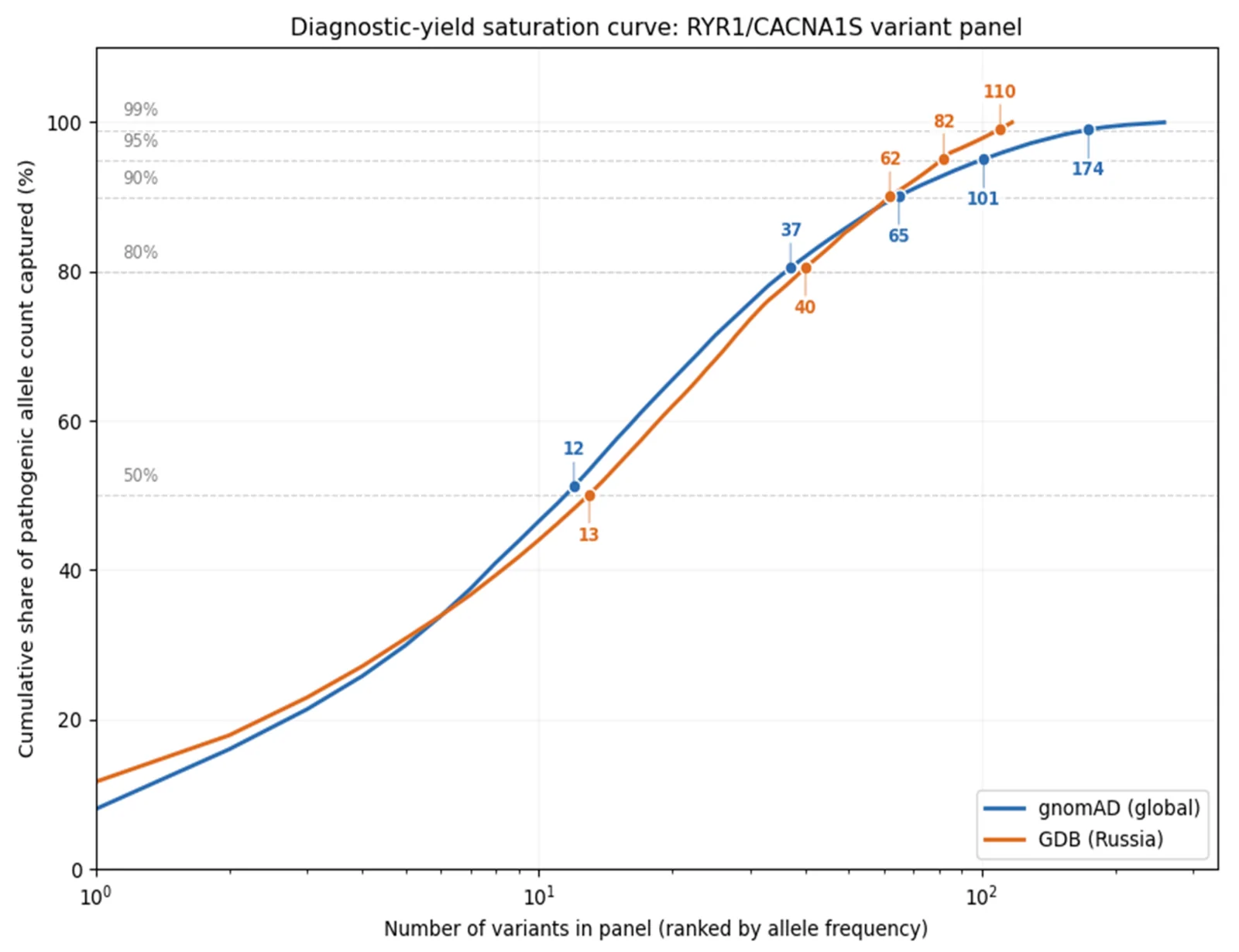
Diagnostic-yield saturation curve for a prioritized *RYR1/CACNA1S* variant panel. Variants were ranked by descending allele count within each population database (gnomAD v4.1.0, blue; GDB v1.3.4, orange), and cumulative allele-count coverage was plotted as a function of panel size (number of top-ranked variants included, log scale). Points mark the minimum panel size required to reach 50%, 80%, 90%, 95%, and 99% cumulative coverage of the total pathogenic allele burden in each database.

To assess how much of the total pathogenic allele burden could be captured by a smaller, prioritized subset of the Core Path variant list, a cumulative allele-frequency saturation analysis was performed separately for gnomAD and GDB (Figure 8). In the global population (gnomAD), the 12 most frequent variants accounted for 50% of the total observed pathogenic allele count, rising to 37 variants for 80% coverage, 65 for 90%, 101 for 95%, and 174 for 99% coverage. In the Russian population (GDB), the corresponding panel sizes were 13, 40, 62, 82, and 110 variants for the same five coverage thresholds.

At lower coverage thresholds (50% and 80%), the panel sizes required were similar between the two populations, with gnomAD requiring marginally fewer variants (12 and 37, respectively) than GDB (13 and 40). At higher coverage thresholds, this pattern reversed: GDB required substantially fewer variants than gnomAD to approach saturation—62 versus 65 variants for 90% coverage, 82 versus 101 for 95%, and 110 versus 174 for 99%. This indicates that the tail of rare, low-frequency contributing variants is proportionally longer in the global gnomAD dataset than in the Russian GDB dataset, consistent with the larger overall sample size and broader population diversity captured by gnomAD. In practical terms, these results suggest that a targeted diagnostic panel of approximately 60–110 prioritized variants could capture the great majority (90–99%) of the pathogenic allele burden observed specifically within the Russian population, without requiring the full 593-variant Core Path reference set.

## 3. Discussion

### 3.1. Genetic Prevalence Versus Clinical Manifestation

It should be emphasized that the estimates reported in Section 2.2 reflect the expected genetic prevalence of pathogenic MHS-associated alleles in the population, rather than the frequency of clinical manifestation. The actual frequency of MH episodes is substantially lower, owing to incomplete penetrance and the requirement for exposure to triggering agents (inhalational anesthetics, succinylcholine). The clinical incidence of MH is best described in terms of the rate per anesthetic procedure, with published estimates of approximately 1 in 10,000 anesthetic procedures in children and approximately 1 in 50,000 in adults. This distinction between genetic carrier frequency and clinically observed incidence is expected for any dominant, incompletely penetrant, trigger-dependent condition, and mirrors the same gap noted between genetics-based and clinically ascertained prevalence estimates for other rare diseases evaluated using similar population-genomic approaches.

An additional consideration is the potential influence of population ancestry on variant frequencies and, consequently, on prevalence estimates. Population-specific or ancestry-enriched variants may contribute disproportionately to observed allele counts in population databases, particularly when their clinical significance remains uncertain. A benign variant enriched in one group can escape frequency-based filtering when only the overall population frequency is considered, since the global estimate is diluted by the larger, predominantly European component of most reference cohorts. This is particularly relevant for *RYR1*, where such variants frequently carry uncertain or historical pathogenic assertions in aggregated databases. *RYR1* c.9242T>C p.(Met3081Thr) is one such case in our data. The variant carries a pathogenic assertion in at least one of the source databases and has an overall frequency of 0.0367% in gnomAD v4.1, below the BS1 threshold of 0.08%; in individuals of African ancestry, however, it reaches 0.6648%, roughly eightfold above the threshold. Variants of this kind are drawn disproportionately from ancestry groups underrepresented in the source literature, so their retention inflates variant sets unevenly across ancestries, and any allele-count-based prevalence estimate inherits the same bias. Applying the BS1 threshold to the maximum ancestry-group frequency rather than to the overall frequency removes such variants at the filtering stage. The remaining differences between groups in our estimates should still be read with ascertainment in mind: expert-curated *RYR1* variant lists derive largely from European-ancestry cohorts, and the lower cumulative frequencies observed in the non-European groups are therefore best treated as lower bounds.

### 3.2. Comparison with Previously Published Genetic Prevalence Estimates

The Core Path Expert estimates obtained here are broadly consistent with previously published genetic prevalence estimates for MHS. Gonsalves et al. reported a prevalence of MHS-associated pathogenic *RYR1* variants of 4 in 870, based on 4 *RYR1* variants [18]. Using genomic databases of *RYR1* and *CACNA1S* variants, another study estimated the prevalence of an MHS-associated pathogenic variant at 1 in 1556, based on 26 unique *RYR1* variants [19]. A more recent population-based exome screening study reported that the frequency of pathogenic or likely pathogenic *RYR1* variants may reach 1 in 600, based on 19 unique *RYR1* variants [20].

Taken together, these findings indicate that the Core Path Expert subset—the most stringently curated of the three tiers evaluated here—yields prevalence estimates most consistent with the existing literature. In contrast, the broader Core Path HGMD and Core Path ClinVar tiers produced substantially higher prevalence estimates, likely reflecting the larger proportion of lower-certainty variants contributed by these sources. As with comparable tiered-pathogenicity frameworks applied to other rare monogenic conditions, this divergence should not be interpreted as evidence that the true prevalence of clinically significant MHS approaches the broader tiers’ estimates; rather, it illustrates how sensitive population-based prevalence modeling is to the evidentiary threshold used to define the causative variant set. A similar tier-based approach was recently applied to population-based prevalence estimation of *KCNV2*-associated retinopathy, where progressively broader variant-evidence categories produced a biologically plausible range of prevalence estimates and highlighted the importance of distinguishing conservative estimates based on well-established pathogenic variants from broader estimates incorporating variants with less certain evidence [21]. This methodological concordance supports the value of reporting a tiered range rather than a single point estimate.

### 3.3. Near-Identical Prevalence Estimates for the HGMD and ClinVar Tiers

Although the Core Path ClinVar tier contains 147 more variants than the Core Path HGMD tier, the two tiers yielded almost identical prevalence estimates in both populations (1 in 104 versus 1 in 102 in Russia; 1 in 50 versus 1 in 50 globally). This indicates that aggregate carrier frequency is driven disproportionately by a small number of relatively common variants already captured within the Core Path HGMD tier, rather than accumulating linearly with the number of curated variants added at each tier. Consistent with this interpretation, the proportion of curated variants represented in at least one population database declined across tiers (65.7% for Core Path Expert, 63.0% for Core Path HGMD, 54.8% for the full Core Path), suggesting that the 147 variants contributed uniquely by ClinVar were represented in population databases at a lower rate than variants in the earlier tiers, and where observed, at correspondingly low individual allele frequencies. Their cumulative contribution to the summed allele count—and thus to the prevalence estimate—was therefore comparatively small, despite their substantial contribution to the total variant count.

### 3.4. Integration of In Silico Predictors, Functional Phenotyping, and ClinGen Criteria

The case study also illustrates the complementary value of in silico pathogenicity predictors and functional phenotyping in the clinical interpretation of *RYR1* variants. Although computational tools such as REVEL can provide supportive evidence regarding the potential functional impact of a variant, in silico predictions alone are insufficient to establish or exclude malignant hyperthermia susceptibility. Phenotypic assessment using the caffeine–halothane contracture test (CHCT), as an established diagnostic assay for MHS, can therefore provide an important additional layer of evidence when interpreting the clinical significance of a variant.

The combination of a negative CHCT result and a low predicted functional impact can provide concordant evidence against a pathogenic effect. Under the ClinGen MHS VCEP specifications, a negative IVCT/CHCT result in a variant-positive individual constitutes phenotype-based evidence against pathogenicity and is applied as BS2; a REVEL score ≤ 0.5 independently supports BP4, which may not be applied in isolation. The *RYR1* variant NM_000540.3:c.418G>A, p.(Ala140Thr) (ClinVar VCV000544412) illustrates how these criteria combine in practice. It was identified in one individual with a negative IVCT/CHCT result (BS2_Moderate), has a REVEL score of 0.261 (BP4_Supporting), and resides within a recognized MHS mutational hotspot (PM1_Moderate); no functional studies have been reported. Bayesian combination of BS2_Moderate, PM1_Moderate and BP4_Supporting yields Likely Benign, the classification submitted for this variant by the VCEP. This illustrates the clinical value of combining phenotype-based evidence with computational predictions: accurate variant reclassification may reduce diagnostic uncertainty and prevent patients from being unnecessarily managed as MHS-susceptible, including the unnecessary use of non-triggering anesthetic protocols and associated clinical restrictions.

### 3.5. Study Limitations

Several limitations should be considered when interpreting these findings.

First, the composition of the curated variant sets depends on database annotations, which differ in evidentiary standards, phenotype specificity, update frequency, and degree of expert review. This limitation is particularly important for HGMD- and ClinVar-derived variants and provides a likely explanation for the substantial increase in inferred susceptibility observed in the expanded tiers.

Second, database assignment of a variant to an MHS-related phenotype does not necessarily establish a definitive causal relationship with MHS. Some variants may have been reported under broad *RYR1*-related disease categories, may have limited functional or segregation evidence, or may have classifications that have not yet been updated in light of more recent evidence. Continued reassessment against primary publications, functional studies, segregation data, and MHS-specific expert classifications will therefore be required.

Third, although both *RYR1* and *CACNA1S* were included in the present framework, the genetic architecture and evidence base are dominated by *RYR1*. The number of well-established MHS-associated *CACNA1S* variants is comparatively small, making *CACNA1S*-specific estimates less stable. In addition, the *RYR1*-specific ClinGen MHS VCEP BS1 threshold of 0.08% was applied to *CACNA1S* for analytical consistency in the absence of an equivalently validated *CACNA1S*-specific threshold. This assumption may affect inclusion of individual *CACNA1S* variants and should be revisited as gene-specific evidence develops.

Fourth, penetrance was not explicitly modeled and was effectively assumed to be complete. Actual penetrance of MHS-associated variants is incomplete and may depend on the specific variant, triggering agent, age, sex, and number of anesthetic exposures. The estimates reported here therefore represent genetic susceptibility rather than expected clinical manifestation.

Fifth, aggregation of allele counts across multiple variants and application of a common population denominator represent approximations. Site-specific allele numbers differ because of variation in sequencing coverage and callability, and the current model does not explicitly account for co-occurrence of multiple qualifying variants within the same individual. These assumptions are expected to have limited impact for individually rare variants but become increasingly relevant as broader variant tiers generate higher aggregate allele frequencies. Future analyses using variant-specific denominators and individual-level genotype data would provide more exact estimates of carrier probability.

Sixth, gnomAD and GDB differ substantially in sample size, ancestry composition, sequencing design, and population representation. Neither dataset should be considered a perfectly representative random sample of its corresponding geographic population. In particular, extrapolation of gnomAD-derived frequencies to the entire world population is illustrative rather than directly epidemiological.

Seventh, population substructure, founder effects, endogamy, and non-random mating may produce regional frequencies that differ considerably from national or global aggregate estimates. This issue is particularly relevant to genetically heterogeneous populations and cannot be fully captured by a single population-wide allele-frequency estimate.

Finally, all variant databases used in this study are dynamic. Variant classifications may change as new functional, segregation, and clinical evidence becomes available. Database versions, retrieval dates, reference transcripts, genomic coordinates, and filtering criteria should therefore be documented to ensure reproducibility and facilitate future updates of the analysis.

## 4. Materials and Methods

### 4.1. Data Sources: Identification and Curation of MHS-Associated Variants

Genetic variants in *RYR1* and *CACNA1S* associated with malignant hyperthermia susceptibility (MHS) were compiled from four complementary sources: ClinGen (Clinical Genome Resource, National Human Genome Research Institute, National Institutes of Health, Bethesda, MD, USA) [22], ClinVar (National Center for Biotechnology Information, National Library of Medicine, National Institutes of Health, Bethesda, MD, USA) [23], HGMD (QIAGEN GmbH, Hilden, Germany) [24], and the European Malignant Hyperthermia Group (EMHG) [25]. *RYR1* and *CACNA1S* represent the two established MHS-associated genes with well-characterized pathogenic variant spectra; database queries were conducted separately for each gene across all four sources, and the resulting variant lists were merged prior to downstream filtering. The use of multiple, methodologically distinct sources was intended to increase the completeness of the curated variant set and the overall sensitivity of the downstream prevalence analysis, since no single database is expected to capture the full spectrum of MHS-associated variants for either gene.

ClinGen is an expert-curated resource in which variants are classified according to strict, standardized clinical criteria; its principal advantage for this study is the high reliability of its interpretations, as submitted data undergo formal expert validation. ClinVar, the largest open archive of clinically significant genetic variants, was used to broaden coverage beyond expert-curated entries. Because ClinVar aggregates independent submissions from numerous laboratories, it can contain discrepancies in interpretation between submitters, and classification evidence may be duplicated across submissions that cite the same underlying publication. In addition, a given variant may be linked in ClinVar both to a specific, narrowly defined disease and to a broader disease group, which complicates unambiguous assignment of the MHS phenotype and requires additional downstream phenotype filtering. HGMD, a commercially licensed database of manually curated disease-associated variants derived from the published literature, was used to capture rare and recently described variants not yet represented in expert-curated resources; however, because HGMD entries may include variants of questionable clinical significance, all HGMD-derived variants were subjected to phenotype matching against the primary literature before inclusion. Finally, EMHG, an international expert body specializing in the study, diagnosis, and interpretation of MHS-associated variants, served as a source of highly specific, causative variants for the disorder and provided an additional layer of clinical specificity not readily available from general-purpose variant databases.

Population-level allele frequencies for the curated *RYR1* and *CACNA1S* variant set were obtained from two complementary resources. The Genome Aggregation Database (gnomAD v4.1.0; Broad Institute of MIT and Harvard, Cambridge, MA, USA) [26] was used as a globally aggregated population genomic resource, comprising sequencing data from 76,000 genomes and 730,000 exomes. The Genetic Diversity Database (GDB v1.3.4; Federal Medical-Biological Agency of Russia, Moscow, Russia) [27] was used as a Russian population-specific resource, comprising sequencing data from 120,000 genomes generated at that center; it enabled assessment of population-specific variant frequencies within the Russian population.

### 4.2. Data Standardization, Merging, and Variant Filtering

After obtaining information from the four source databases (EMHG, HGMD, ClinVar, and ClinGen), variant records were standardized and merged into a single non-redundant dataset. All variants were converted to a unified chromosome-position-reference-alternate (chr-pos-ref-alt) or VCF-compatible format to ensure correct cross-database matching, and duplicate entries were removed to avoid double counting of variants reported by more than one source. This step yielded a combined pool of 13,918 unique reported variants.

From this pool, each source was then curated independently, using criteria appropriate to its data structure, before the resulting subsets were reconciled into a final reference set (Figure 1). Two sources were treated as expert-curated and were carried forward without additional filtering beyond their own classification: variants listed in EMHG were retained as reported, and variants in ClinGen were retained if they carried a pathogenic/likely pathogenic (P/LP) verdict from the ClinGen MHS Variant Curation Expert Panel (MHS VCEP). These two subsets were combined into the Core Path Expert set, representing the most stringently curated tier of the reference variant list.

The HGMD- and ClinVar-derived variants required more extensive filtering, since both sources aggregate submissions of variable specificity and evidence quality. HGMD entries were first restricted to variants carrying a DM or DM? tag (disease-causing or likely disease-causing), then filtered in for an MHS-related phenotype based on the titles of the citing publications or the disease term under which the variant was submitted and finally filtered out if they carried a conflicting benign/likely benign (B/LB) classification in ClinVar. ClinVar entries were independently restricted to variants with a P/LP verdict and filtered out if the only phenotype associated with the variant was an autosomal recessive (AR) myopathy, which is inconsistent with the predominantly autosomal dominant inheritance of MHS.

The variants surviving these source-specific filters from HGMD and ClinVar were then pooled and subjected to a shared final filtering stage: loss-of-function variants (frameshift, stop-gain, and splice-site–disrupting changes), intronic variants, and synonymous variants were excluded, and the remaining variants were retained only if their population allele frequency did not exceed 0.08%, corresponding to the BS1 frequency threshold defined in the ClinGen MHS VCEP specifications. The threshold was applied to the maximum allele frequency observed across the AFR, AMR, EAS, NFE and SAS genetic ancestry groups of gnomAD v4.1, rather than to the overall frequency, so that variants common in a single ancestry group would not escape the filter. The AMI, ASJ, FIN, MID and remaining groups were excluded from this maximum, consistent with the gnomAD grpmax convention, as founder effects and small sample sizes render frequency estimates in these groups unstable. Variants removed by this criterion that would have passed a filter based on the overall frequency are listed in Supplementary Table S4. Allele frequencies were obtained from the gnomAD v4.1 joint exome and genome release. Only variants with a FILTER value of PASS were retained; in the joint release this value indicates that quality filters were not triggered in either the exome or the genome callset, whereas the EXOMES_FILTERED, GENOMES_FILTERED and BOTH_FILTERED categories flag variants failing quality control in one or both data types. Applying this criterion excluded four variants with very low allele frequency, and the resulting prevalence estimates were essentially unaffected. Variants with a joint allele count of zero were likewise excluded, as no allele was observed in the cohort. Variants passing this combined filter were then attributed back to their source of origin, yielding the Core Path HGMD and Core Path ClinVar subsets.

The three source-specific subsets—Core Path Expert, Core Path HGMD, and Core Path ClinVar—were subsequently merged (union) into a single core pathogenic reference set comprising 593 unique variants (Core Path), which formed the basis for prevalence estimation.

### 4.3. Prevalence Estimation

Prevalence of MHS was estimated using the Hardy–Weinberg equilibrium model, which relates observed allele frequencies to expected genotype frequencies in the population. Because MHS follows an autosomal dominant inheritance pattern, the condition manifests in carriers of at least one copy of a pathogenic allele—that is, in both heterozygotes and homozygotes for the pathogenic allele. Accounting for the shared probability space between the two allele copies at a locus to avoid double-counting homozygous carriers yields the exact relationship:

Prevalence = 2pq + q^2^ = 1 − (1 − q)^2^ = 2q − q^2^

where q is the aggregate frequency of pathogenic MHS alleles.

Rather than treating q as a fixed value obtained from a point estimate, allele frequency was modeled as a random variable and estimated using a Bayesian framework adapted from the approach developed by Schrodi et al. for autosomal recessive disease prevalence estimation from population-based genetic data [28]. For each variant set, the observed allele count (AC) among the total number of alleles evaluated (AN) defines the posterior distribution of the underlying allele frequency Q under a Beta-binomial conjugate model:

Q|AC, AN ∼ Beta(AC, AN − AC)

The point estimate of prevalence was calculated as the posterior expectation of the exact dominant-model formula, E[2Q − Q^2^], obtained in closed form from the first and second moments of this Beta distribution:

E[Q] = AC/AN

E[Q^2^] = AC(AC + 1)/[AN(AN + 1)]

E[Prevalence] = 2 · E[Q] − E[Q^2^]

The 95% credible interval was derived from the same posterior distribution used for the point estimate. The lower and upper bounds of the interval for Q were obtained as the 2.5th and 97.5th percentiles of Beta(AC, AN − AC):

q_low = Beta^−1^(α/2, AC, AN − AC)

q_high = Beta^−1^(1 − α/2, AC, AN − AC)

where α denotes the significance level (0.05 for the 95% interval). Because the transformation g(q) = 2q − q^2^ is monotonically increasing over the relevant range, these bounds were propagated directly to the prevalence scale without requiring simulation:

Prevalence_low = 2 · q_low − q_low^2^

Prevalence_high = 2 · q_high − q_high^2^

Using a single posterior distribution for both the point estimate and the credible interval keeps the estimator internally consistent. A penetrance term was retained in the model to allow for future refinement but was set to 1 (complete penetrance) throughout the current analysis, in the absence of MHS-specific penetrance estimates.

For each variant set, AC was calculated as the sum of allele counts across all constituent variants, while AN was taken as the maximum reported allele number among those variants within the corresponding database, representing the total number of alleles genotyped in that population sample (AN varies only marginally across sites within a given database, reflecting site-specific differences in call rate, rather than differing denominators to be summed).

This procedure was applied separately to three nested variant sets, reflecting the source-specific curation tiers and yielding an increasingly inclusive series of prevalence estimates: Prevalence 1st, calculated from the Core Path Expert set alone (EMHG and ClinGen MHS VCEP); Prevalence 2nd, calculated after adding Core Path HGMD variants to the Core Path Expert set; and Prevalence 3rd, calculated after further adding Core Path ClinVar variants, corresponding to the full Core Path reference set. When HGMD- and ClinVar-derived variants were incorporated into the 2nd and 3rd tiers, the population allele frequency threshold of 0.08% was reapplied at the level of each specific population database, so that a variant contributed to a given tier only if its allele frequency in that database (gnomAD or GDB individually) also satisfied this threshold. Each of the three tiers was evaluated independently against gnomAD v4.1.0 and GDB v1.3.4, allowing comparison of global and Russian population-based estimates at each level of curation stringency.

To express results in absolute terms, the point estimate and credible interval of prevalence for each tier were additionally scaled by an estimate of the corresponding reference population size—approximately 8 billion individuals for the global population (evaluated against gnomAD) and approximately 146 million individuals for the Russian Federation (evaluated against GDB)—to project the estimated number of affected individuals in each population, alongside the “1 in N” prevalence format.

## 5. Conclusions

Population-based estimation of malignant hyperthermia susceptibility is strongly dependent on the evidentiary criteria used to define causative variants. In the present analysis, the high-confidence Expert tier, based on EMHG and ClinGen MHS VCEP classifications, yielded genetically inferred susceptibility frequencies of approximately 1 in 807 in gnomAD and 1 in 1078 in the Russian GDB dataset, broadly consistent with previously reported population-genomic estimates.

Progressive inclusion of HGMD- and ClinVar-derived variants increased the estimated frequencies to approximately 1 in 63–65 in gnomAD and 1 in 102–104 in GDB. These expanded estimates should be interpreted as sensitivity analyses and approximate upper bounds of database-defined MHS-associated variant burden rather than as estimates of clinically penetrant malignant hyperthermia. The marked divergence between the Expert and expanded tiers demonstrates that variant-classification stringency is a major determinant of population-based prevalence estimates. This marked shift also serves as a caution against relying on uncurated or insufficiently curated mutation databases for population-genetic prevalence estimation, as inclusion of such variants may substantially overestimate the apparent burden of MHS-associated alleles.

The analysis also identified substantial population-specific differences in the distribution of MHS-associated alleles and showed that a relatively limited number of recurrent variants account for most of the observed allele burden, whereas a long tail of rare variants contributes increasingly at higher coverage thresholds.

Overall, these findings support a tiered framework for estimating MHS genetic susceptibility that explicitly distinguishes high-confidence expert-curated variants from broader database-derived candidate variants. Future refinement incorporating variant-specific penetrance, gene-specific frequency thresholds, functional evidence, individual-level genotype data, and population-specific genomic resources will be necessary to bridge the gap between genetically inferred susceptibility and the clinically observed incidence of malignant hyperthermia.

## Figures and Tables

**Table 1:** Calculated prevalence based on core pathogenic variants.

Characteristics	Core Path Expert	Credidle Interval
gnomAD v4.1.0		
Affected 1/over	1/807	1/759–1/859
Affected globally	9,918,654 affected people	9,314,005–10,542,027 affected people
GDB v1.3.4		
Affected 1/over	1/1078	1/904–1/1310
Affected in Russia	135,375 affected people	111,477–161,557 affected people

**Table 2:** Prevalence estimates across expanded variant tiers.

Characteristics	Core Path HGMD	Core Path ClinVar
	gnomAD v4.1.0	
Affected 1/over	1/65	1/63
Credible interval	1/64–1/66	1/62–1/64
Affected globally	123,810,356 affected people	126,632,673 affected people
Credible interval	121,669,298–125,969,539 affected people	124,467,725–128,815,729 affected people
	GDB v1.3.4	
Affected 1/over	1/104	1/102
Credible interval	1/98–1/110	1/96–1/108
Affected in Russia	1,401,464 affected people	1,432,744 affected people
Credible interval	1,322,398–1,482,770 affected people	1,352,799–1,514,929 affected people

## Data Availability

Publicly available databases: gnomAD v4.1.0 (https://gnomad.broadinstitute.org/, accessed on 31 July 2026), ClinVar (https://www.ncbi.nlm.nih.gov/clinvar/, accessed on 31 July 2026), ClinGen (https://erepo.clinicalgenome.org/redmine/projects/evrepo/pcer/, accessed on 31 July 2026), EMHG (https://www.emhg.org/diagnostic-mutations, accessed on 31 July 2026). Restricted-access databases: HGMD (https://digitalinsights.qiagen.com/, accessed on 31 July 2026), GDB FMBA v1.3.4 (https://gdbpop.nir.cspfmba.ru/, accessed on 31 July 2026). Derived datasets provided in this study: We provide Core Path ClinVar preprocessed dataset containing *RYR1* and *CACNA1S* variants. All variants in this dataset are de-identified: information regarding the specific source database for each variant has been removed. This approach respects the licensing requirements of the original databases, including HGMD, whose data cannot be redistributed without explicit permission. Additionally, we provide the list of variants that exceeded the BS1 threshold in at least one genetic ancestry group and were therefore excluded from the analysis.

## Supplementary Materials

The following supporting information can be downloaded at: https://www.mdpi.com/article/10.3390/ijms27188327/s1.

## Abbreviations

The following abbreviations are used in this manuscript:

MHS	Malignant hyperthermia susceptibility
MH	Malignant hyperthermia
CK	Creatine kinase
DHPR	Dihydropyridine receptor
VUS	Variants of uncertain significance
VCEP	Variant Curation Expert Panel
P	Pathogenic
LP	Likely pathogenic
B/LB	Benign/likely benign
LoF	Loss of function
IVCT	In vitro contracture test
CHCT	Caffeine-halothane contracture test
EMHG	European Malignant Hyperthermia Group
FMBA	Federal Medical-Biological Agency

## References

[1] Riazi S., Biesecker L.G., Rosenberg H., Dirksen R.T., Adam M.P., Bick S., Mirzaa G.M., Wallace S.E., Amemiya A. (2003). Nonsyndromic Malignant Hyperthermia Susceptibility. GeneReviews^®^ [Internet].

[2] Lebedinskii K.M., Prokopiev G.G., Stepanenko S.M., Dunts P.V., Molchanov I.V., Ovechkin A.M., Alexandrovich Y.S., Lomivorotov V.V., Shen N.P., Trembach A.V. (2023). Malignant hyperthermia due to anaesthesia. Russ. J. Anesthesiol. Reanimatol..

[3] Tukhvatullina R.R., Matinyan N.V. (2023). Malignant hyperthermia (literature review). Messenger Anesthesiol. Resusc..

[4] Kim D.C. (2012). Malignant hyperthermia. Korean J. Anesthesiol..

[5] Yu K.D., Betts M.N., Urban G.M., Schwartz M.L.B., Robinson T.O., Moyer R.J., Taddonio S.W., Vasudevan A., Johns A., Sturm A.C. (2024). Evaluation of Malignant Hyperthermia Features in Patients with Pathogenic or Likely Pathogenic RYR1 Variants Disclosed through a Population Genomic Screening Program. Anesthesiology.

[6] Johnston J.J., Dirksen R.T., Girard T., Gonsalves S.G., Hopkins P.M., Riazi S., Saddic L.A., Sambuughin N., Saxena R., Stowell K. (2021). Variant curation expert panel recommendations for RYR1 pathogenicity classifications in malignant hyperthermia susceptibility. Genet. Med..

[7] Stephens J., Schiemann A.H., Roesl C., Miller D., Massey S., Pollock N., Bulger T., Stowell K. (2016). Functional analysis of RYR1 variants linked to malignant hyperthermia. Temperature.

[8] European Malignant Hyperthermia Group EMHG Homepage. http://www.emhg.org/home/.

[9] Rossi D., Pranzo C., Roccabianca S., Galli L., Orrico A., Sorbello G., Tegazzin V., D’Onofrio P., Fucci A., Quarta S. (2026). Identification of novel potentially causative RYR1 variants in individuals with malignant hyperthermia susceptibility. Neuromuscul. Disord..

[10] Johnston J.J., Dirksen R.T., Girard T., Hopkins P.M., Kraeva N., Ognoon M., Radenbaugh K.B., Riazi S., Robinson R.L., Saddic I.L.A. (2022). Updated variant curation expert panel criteria and pathogenicity classifications for 251 variants for RYR1-related malignant hyperthermia susceptibility. Hum. Mol. Genet..

[11] McCarthy T.V., Quane K.A., Lynch P.J. (2000). Ryanodine receptor mutations in malignant hyperthermia and central core disease. Hum. Mutat..

[12] Monnier N., Ferreiro A., Marty I., Labarre-Vila A., Mezin P., Lunardi J. (2003). A homozygous splicing mutation causing a depletion of skeletal muscle RYR1 is associated with multi-minicore disease congenital myopathy with ophthalmoplegia. Hum. Mol. Genet..

[13] Sarıkaya Uzan G., Özyılmaz B., Doğan G., Baydan F., Güzin Y., Gençpınar P., Gazeteci Tekin H., Olgaç Dündar N. (2026). Clinical and Genetic Spectrum of RYR1-Related Disease. Mol. Syndromol..

[14] Guo W., Sun B., Estillore J.P., Wang R., Chen S.R.W. (2020). The central domain of cardiac ryanodine receptor governs channel activation, regulation, and stability. J. Biol. Chem..

[15] Xiao Z., Guo W., Sun B., Hunt D.J., Wei J., Liu Y., Wang Y., Wang R., Jones P.P., Back T.G. (2016). Enhanced Cytosolic Ca^2+^ Activation Underlies a Common Defect of Central Domain Cardiac Ryanodine Receptor Mutations Linked to Arrhythmias. J. Biol. Chem..

[16] de Mello J.M., Andrade P.V., Santos J.M., Oliveira A.S.B., Vainzof M., do Amaral J.L.G., da Silva H.C.A. (2023). Predictive factors of the contracture test for diagnosing malignant hyperthermia in a Brazilian population sample: A retrospective observational study. Braz. J. Anesthesiol..

[17] Girard T., Treves S., Voronkov E., Siegemund M., Urwyler A. (2004). Molecular genetic testing for malignant hyperthermia susceptibility. Anesthesiology.

[18] Gonsalves S.G., Ng D., Johnston J.J., Teer J.K., Stenson P.D., Cooper D.N., Mullikin J.C., Biesecker L.G., NISC Comparative Sequencing Program (2013). Using exome data to identify malignant hyperthermia susceptibility mutations. Anesthesiology.

[19] Mungunsukh O., Deuster P., Muldoon S., O’Connor F., Sambuughin N. (2019). Estimating prevalence of malignant hyperthermia susceptibility through population genomics data. Br. J. Anaesth..

[20] Kelly M.A., Leader J.B., Wain K.E., Bodian D., Oetjens M.T., Ledbetter D.H., Martin C.L., Strande N.T. (2021). Leveraging population-based exome screening to impact clinical care: The evolution of variant assessment in the Geisinger MyCode research project. Am. J. Med. Genet. C Semin. Med. Genet..

[21] Rozhkova A.V., Rutkovskaya E.A., Kadyshev V.V., Esibov A.A., Alsalloum A., Kuznetsova E.S., Krupinova J.A., Mityaeva O.N., Shefer K.K., Boiko E.V. (2026). Bayesian Population-Based Prevalence Estimation of KCNV2-Associated Retinopathy: A Comparative Analysis of Disease-Associated Variant Frequencies in Russian and Global Populations. Int. J. Mol. Sci..

[22] Rehm H.L., Berg J.S., Brooks L.D., Bustamante C.D., Evans J.P., Landrum M.J., Ledbetter D.H., Maglott D.R., Martin C.L., Nussbaum R.L. (2015). ClinGen—The Clinical Genome Resource. N. Engl. J. Med..

[23] Landrum M.J., Lee J.M., Benson M., Brown G., Chao C., Chitipiralla S., Gu B., Hart J., Hoffman D., Hoover J. (2016). ClinVar: Public Archive of Interpretations of Clinically Relevant Variants. Nucleic Acids Res..

[24] Stenson P.D., Mort M., Ball E.V., Chapman M., Evans K., Azevedo L., Hayden M., Heywood S., Millar D.S., Phillips A.D. (2020). The Human Gene Mutation Database (HGMD^®^): Optimizing Its Use in a Clinical Diagnostic or Research Setting. Hum. Genet..

[25] European Malignant Hyperthermia Group (EMHG) (Likely) Pathogenic Mutations. https://www.emhg.org/diagnostic-mutations.

[26] Chen S., Francioli L.C., Goodrich J.K., Collins R.L., Kanai M., Wang Q., Alföldi J., Watts N.A., Vittal C., Gauthier L.D. (2024). A Genomic Mutational Constraint Map Using Variation in 76,156 Human Genomes. Nature.

[27] Database of Population Frequencies of Genetic Variants of the Population of the Russian Federation. Federal Medical-Biological Agency of Russia. https://gdbpop.nir.cspfmba.ru/.

[28] Schrodi S.J., DeBarber A., He M., Ye Z., Peissig P., Van Wormer J.J., Haws R., Brilliant M.H., Steiner R.D. (2015). Prevalence Estimation for Monogenic Autosomal Recessive Diseases Using Population-Based Genetic Data. Hum. Genet..

